# Proceedings of the Medical Convention of Ohio, Held at Columbus, on the 5th, 6th, and 7th of May, 1841: With Several Papers Read before That Body

**Published:** 1842-05

**Authors:** 


ART. IV.—*Proceedings of the Medical Convention of Ohio, held at Columbus, on the 5th, 6th, and 7th of May,* 1841: *with several papers read before that body.* Columbus, Wright and Legg, 1841, pp. 84.

As originally proposed, this convention assembled every third year. At the second triennial session, held at Columbus in January, 1838, an adjournment was made to May 1839, and it was recommended that the meetings thereafter should be held annually in that month. An interval, howev-

---

er, of two years occurred between that session and the one the proceedings of which we have before us.

The number in attendance at the last meeting, does not appear to have been so great as in the preceding years; about fifty persons only were present, representing fifteen counties of the State. Dr. George W. Boerstler was chosen President; Drs. Russel, Fisher, Harrison, Howard, Miller and Steele, Vice-Presidents; Dr. Kreider, Recording Secretary; Dr. Mendenhall, Corresponding Sec'y.; and Dr. Thompson, Treasurer.

The Journal of the convention does not exhibit a large amount of business, owing partly, no doubt, to the small number of members present, and partly to the circumstance that all the committees, save one, appointed at the last session, failed to make reports. Papers were presented by Drs. Harrison, Dawson and Bigelow, and a report on Auscultation and Percussion submitted by Drs. Mendenhall, Howard and Brayton. The reading and discussion of these papers, with the passage of sundry resolutions instructing divers committees to investigate a variety of subjects therein named, and make reports at the next meeting, constituted the chief exercises of the convention. We say *chief*, because a portion of time was occupied in listening to lectures on *tight-lacing*, *phrenology and quackery*. You smile, gentle reader! at such a conjunction of most dissimilar matters. Nevertheless it is true. The very first thing done in convention, after the body was organized, was the adoption of the following resolutions:

*Resolved*, That Professor Mussey be invited to deliver a discourse upon the subject of "Tight Lacing," at such time as may suit his convenience, during the session of this Convention.

*Resolved*, That Professor Mussey be invited to deliver a discourse upon the subject of Phrenology, at such time and place as may be designated, during the present session of the Convention.

And on the morning of the second day we have this:

*Resolved*, That Dr. J. P. Harrison be requested to deliver a lecture on the subject of quackery and empiricism, during the sitting of the Convention.

We sincerely hope the members were benefited by these discourses; yet the idea of such "grave and reverend signiors" sitting in wrapt attention—*arrectis auribus*, to speak pedantically—under lectures on tight-lacing and quackery (we say nothing of phrenology), smacks a little of the absurd, and is somewhat laughter-moving withal. Are there no quacks and empirics in all the fifteen counties whence these gentlemen came, that they must needs have the creatures described? Verily, it would be strange if such omnipresent bipeds were not to be found in those regions somewhere! Are *corsets* growing more in favor there, or are the *laces* drawn tighter than formerly, that the convention must visit them with their objurgations? Truly, we did suppose that the "buckeye ladies" (whom we learned to prize, what time we dwelt north of the Ohio), with their characteristic good sense, had altogether eschewed such fearsome things! If, though, it be otherwise, and the keen sarcasm and massive argument of the eloquent lecturer (who, as some of our readers wot, wields with equal readiness the light blade of the Soldan and the ponderous mace of him of the Lion-heart), be insufficient to loose the said strings, we beg the convention to try the virtue of the famous composition of Ernulphus the bishop, and that the same be read "with good accent and good discretion" at the next meeting, adding uncle Toby's *Lillibullero* as a running accompaniment, if they choose. Badinage aside, though, it seems to us that such an assemblage is not suitable for matters of this kind. Popular lectures, even on well-worn subjects, may be good in their place, but surely that place is not a medical convention. Neither is it any better suited to demonstrations in anatomy; and we pray, therefore, that Professor Shotwell may *not* "prepare a subject and demonstrate the surgical anatomy of Hernia to the medical convention at its next meeting," as he is requested to do. If elementary instruction of this kind, or any other, be required by members, we suggest the propriety of their attending the prelections of that gentleman and his colleagues the ensuing winter.

The papers read to the convention, and printed with the proceedings, are not without interest, and give evidence of what such an association may do, if its members are imbued with the proper spirit. Two of them are from the ready pen of Professor Harrison of the Medical College of Ohio. The one is a sketch of the diseases induced by mercury, the other is an essay on medical education. In the former, Prof. H. gives a rapid view of the various morbid effects resulting from the inordinate or inappropriate use of mercury, with the treatment applicable thereto. He divides these effects into the immediate and the remote. The former are those arising from "its violent action on the stomach, bowels, mouth, skin, and nervous system." The latter embrace "palsy, ulcerations of the soft parts, and diseases of the bones." He contends strenuously, in opposition to Musgrave, Carmichael, Chapman and others, that mercury does "produce diseases of the throat, skin and bones, with their investing membranes, analogous in aspect to those of an unequivocal venereal origin, and equally destructive in their progress." In support of this opinion, he adduces several cases derived partly from his own experience, which certainly go far towards substantiating it. He admits, however, that these diseases have no distinctive and peculiar marks whereby they may be distinguished from venereal affections of the same nature. In their treatment he relies chiefly on arsenic, in the form of Fowler's solution, and sarsaparilla. Narcotics will be required to abate morbid irritability, and where a cachectic state co-exists with this, as we presume it does in most instances (for it is doubtful whether mercury would produce the effects under consideration in any other than a depraved habit of body), tonics alone or combined with narcotics will be found to fulfil the indication better. The hydriodate of potash combined with the iodide of iron, given in pretty large doses, has of late years been used with remarkable success in these cases. As a local application to the ulcerated surfaces, the professor recommends a mixture of sulphate of copper, Peruvian bark, and balsam copaiva, which he also employs in the ordinary mercurial sore-mouth. Attention must be paid to regimen; the diet should be bland and nutritious, tea, coffee, and all stimulating drinks being avoided; flannel or cotton should be worn next the skin; a regulated temperature should be maintained in the apartment, and the patient must avoid a damp cold atmosphere.

The subject of medical education, although a very trite one, Prof. H. treats with his usual ability, and he has succeeded in making a very interesting essay out of it. His views on the subject are perfectly orthodox, and such as will meet with the entire concurrence of all who have the good of the profession at heart. He insists upon a high standard of qualifications, both as a preparation for the study of medicine and for the discharge of the responsible duties of a medical practitioner. To bring about the reforms which he conceives to be necessary, two modes are pointed out: 1st. That physicians should not receive as pupils young men who are deficient in moral or intellectual qualifications: 2nd. That the sessions of our medical schools should be prolonged, the number of chairs increased, and a greater length of time be devoted to the acquisition of a strictly professional education. Recommendations similar to these were promulgated ten years ago in an "Essay on Medical Education," from the pen of the senior editor of this journal, who at that time filled the chair of Theory and Practice in the medical college of our sister State. The following resolutions pertaining to the same subject, and apparently called forth by the reading of the essay, were adopted by the Convention:

*Resolved*, That the system of Medical Education in the United States is radically defective.

*Resolved*, That the evil can alone be remedied by those having charge of medical schools.

*Resolved*, That it be respectfully, but earnestly, recommended to the Trustees and Professors of the medical schools in the United States, to meet in Convention, at such time and place as may be agreed upon by themselves, to digest and recommend a general system of Medical Education.

Similar resolutions were passed at the second session of this body, in 1838. Subsequent to that period, the Medical Society of the State of New York named the 12th of May, 1840, and the city of Philadelphia, as a proper time and place for a general meeting. This meeting proved a failure, as very few of the schools selected representatives to attend. (See West. Journal, vol. I., p. 366). The indifference and apathy manifested in relation to this subject, is truly a matter of astonishment. All concede the necessity of reform, and to secure it, a little concert of action would be amply sufficient. Indeed, it is in the power of one single school to effect this change; and that school is the University of Pennsylvania. Standing, as she confessedly does, at the head of similar institutions in this country, as well by her age as the long line of illustrious names that have graced her halls, she is bound by every tie of duty to herself, and the profession, to set the example. No one can doubt that if she were to do this, the desired reformation would instantly go forward. But so long as she withholds her assent, or refuses to take any decided step in the matter, it will be in vain to expect any alteration in the existing state of things.

We cannot, however, entirely agree with the second resolution quoted above. It is not in the power of medical schools *alone* to effect the reform. That much may be done by proper action on their part, with the other measures proposed, is freely admitted; yet this will not wholly guard against the admission of unworthy men into the profession. One source of evil lies deeper still; society at last must aid in applying the corrective. So long as people look to the *man* rather than his *skill* and *attainments*, and suffer themselves to be cajoled and wheedled; so long as individuals are found who will court and flatter, and

> "—— bend the pregnant hinges of the knee
> That thrift may follow fawning,"

so long will our noble profession be disgraced by base and miserable pretenders.

The "Report on Auscultation and Percussion" is short and pertinent. It sets forth in a few words the necessity of accurate diagnosis to the successful treatment of disease, and replies to the objections that have been raised against physical exploration, by showing its usefulness in affections of the lungs, heart and abdominal viscera, and its importance in the diagnosis of pregnancy, &c. It asserts the practicability of physicians in country practice becoming skilful in the application of this means, and concludes with the following excellent sentiment: "it behooves every conscientious physician to avail himself of all the means in his power, by which certainty of doing good is added to the present scanty resources of our art."

The fourth article, entitled "Florula Lancastriensis," is a catalogue of the flowering and filicoid plants indigenous to Fairfield county, Ohio, accompanied by notes of such as are medicinal. More than 400 plants are contained in this list, which seems to have been drawn up with great care and labor. The author, Dr. Bigelow, urges upon his professional brethren the importance of the study of botany; he says he has found within the limits of his own county, more than one hundred and ninety species of plants possessing medicinal properties, less than one half of which have their medicinal histories reduced to anything like certainty. We commend the following remarks to the notice of our readers.

"With a little care and attention, every physician could become tolerably well acquainted with the local botany within the sphere of his daily avocations, and an enumeration of the species of plants from all quarters of the State, would be of great value to systematic botanists in affixing the precise limits and localities to our more valuable ones."

"There are many points in the study of this fascinating science, in which the enlightened practitioner of medicine must feel an abiding interest, particularly those points having reference to the anatomy and physiology of plants, and a faithful inquiry into their sensible and medicinal properties."

These considerations are certainly sufficient to entitle this science to a claim on our attention. But, apart from its interest in this respect, it has attractions that are peculiar to itself. It opens one of the purest sources of enjoyment that can be offered to the mind. Witness the ardor and devotion with which the botanist pursues his researches through all changes of time and seasons, never wearying, never tiring, and when compelled to desist, burning with the same zeal as when he began. He seems to live in a little world of his own—a beautiful and ever-varying one, too, wherein nothing profane has ever entered, but which continues as bright and pure as when first called into existence by the Great Author of life.

The fifth and last paper is from the pen of Dr. Dawson, whose name is familiar to the readers of this journal. It relates to a form of fever that prevailed in the eastern part of Green County, Ohio, where the author resides, and bears the impress of an acute and inquiring mind. The season that preceded this epidemic was of an unusual kind, exhibiting the extremes of humidity and drought. Its effect on the health of the inhabitants was evinced rather by a change in the character of the ordinary diseases, than by any unusual amount of sickness. Remittent and intermittent fevers were more prevalent than formerly, and the latter visited sections of country previously exempt from it. The epidemic occurred both in winter and summer; it received a variety of names. Some called it typhus from its resemblance to that affection.

"*Symptoms.*—The onset of the disease was manifested by pain in the head, sometimes confined to a circumscribed spot; severe cases did occur, however, in which the cerebral distress was so inconsiderable as to be unnoticed. Some complained of chills in the commencement, though it was by no means a pathognomic of the disease.

The tongue was generally covered with a *thin white* fur, through which the papilla protruded, the sides and apex being red, and the shape not much changed from the normal. There was no appreciable abdominal tenderness in the commencement, but it generally supervened after a few days had elapsed, and with it protracted discharges from the bowels. The sanguiferous system was but little disturbed—the pulse *small* and *soft*, and seldom exceeded 80 beats to the minute. Skin *soft* and *natural*, except a slight elevation of temperature. Rose colored spots *occasionally* made their appearance over the whole surface, but always on the cheeks. They would make their appearance and recede again several times in the course of twenty-four hours. A dry cough attended most cases."

"This description seems to present no formidable array of symptoms; yet it is a fact, that the disease under the best directed efforts of the physician, did sometimes last fifty days. Few cases recovered under twenty days, and it was not uncommon to see convalescence protracted to four or five weeks."

"Much evidence might be cited to prove that it was contagious, as much so as scarlatina, and generated and propogated in a similar manner."

Concerning the pathology of this disease, the author offers nothing but conjecture; as, owing to the circumstances surrounding him, he was precluded from making autopsic examinations. From the character of the symptoms, however, and the kind of treatment most successful, he infers that the principal lesion consisted in irritation of the mucous membrane of the alimentary canal, which, in protracted cases, ran into ulceration.

The treatment that proved most successful "was a modification of the physiological." General blood-letting was of doubtful value, and was employed with hesitation in consequence of the little disturbance of the sanguiferous system. Nevertheless the author's personal experience leads him to believe that in most cases where this remedy was omitted, local inflammation was apt to supervene, and that the course of the fever was more protracted. Mild purgatives were useful throughout the whole course of the disease. Emetics, though they produced their physiological effects promptly, did not exert any favorable influence on the malady. Ptisans, mucilaginous drinks, and the water of sub-acid fruits, were found serviceable in all stages. Indeed these, with laxatives, and stimulants in the latter stages (wine-whey proved the most useful), were the chief remedies employed.

"When the main force of the fever was subdued, it was generally a matter of much solicitude with the physician to see the approach of convalescence. His expectations, however sanguine, were doomed to disappointment. It was not uncommon for the feverin this stage, to remit. These remissions occupied the forenoon, lasting five or six hours. In the afternoon the exacerbations took place. These remissions and exacerbations were quotidian, and would sometimes last four or five weeks, to harass the patient and mortify the physician. Antiperiodics seemed to be somewhat indicated when the fever assumed this form, but they answered no curative purpose at all. On the contrary, they aggravated the malady by increasing the exacerbation."

The author supposes this condition to have depended on morbid alterations in the alimentary canal; it was treated by "infusions of slippery-elm and gum-water."

The next meeting of the Convention, as was stated in our last, will be held on the 16th inst., in Cincinnati. We trust that it will be well attended. Associations of this kind are calculated to do a vast amount of good. The interchange of opinion which they invite, is essential to the improvement, if not to the very existence of our science; by it "time-honored" errors are corrected, new truths elicited, and a spirit of inquiry aroused. But they do more. They bring together individuals from various quarters of the country, and thus serve to break down those sectional prejudices and feelings which are the source of so much discord among medical men. They diffuse a spirit of emulation through the ranks of the profession, and thus elevate its character and dignity. They inspire sentiments of mutual respect and esteem among its members, and thus tend to soften the asperities that so often mark their intercourse. This consideration alone should lead us to regard them favorably; for it is painful to see men, whose daily avocations call into exercise the finer feelings of the heart, exhibit, as they frequently do, in their conduct towards each other, so much of the bitterness and gall that pertain to our fallen nature. C.

