# Erratum

**Published:** 1842-06

**Authors:** 


### ERRATUM.

In page 363 of the last number,12th line from the top, in the article on Dr. Bigelow's *Florula Lancastriensis,* after "400" read "genera of."

